# Sustainable financing options for mental health care in South Africa: findings from a situation analysis and key informant interviews

**DOI:** 10.1186/s13033-019-0260-4

**Published:** 2019-01-21

**Authors:** Sumaiyah Docrat, Crick Lund, Dan Chisholm

**Affiliations:** 1 Alan J Flisher Centre for Public Mental Health, Department of Psychiatry and Mental Health, University of Cape Town, Cape Town, South Africa; 2 Centre for Global Mental Health, Institute of Psychiatry, Psychology and Neuroscience, King’s College London, London, UK; 3 Department of Mental Health and Substance Abuse, World Health Organization, Geneva, Switzerland

**Keywords:** Mental health systems, Healthcare financing, Sustainable mental health financing, National Health Programs, Health Care Reform, Global Mental Health

## Abstract

**Background:**

With the implicit neglect for the integration of mental health services into general health service development in South Africa, there is an urgent need for an understanding of the ways in which existing reforms may be leveraged to incorporate the objectives of the *National Mental Health Policy Framework and Strategic Plan* (MHPF) and the mechanisms by which these reforms can be structured and financed in the context of fiscal constraint.

**Methods:**

A situational analysis guided by a newly developed analytical framework for sustainable mental health financing was conducted. The review was followed by qualitative, indepth interviews with a range of expert national stakeholders.

**Results:**

Although the MHPF is said to be consistent with ongoing efforts toward the implementation of National Health Insurance (NHI), there is clear evidence of discordance between the MHPF and the NHI. The most promising strategies for sustainable mental health financing include: increased decentralization of resources to primary and community mental health services; active integration of mental health into ongoing NHI implementation including expanding the mandate of District hospitals and drawing on the private sector; submission of costed budget bids to support a mental health conditional grant and ensuring that explicit outcomes and deliverables are in place to monitor Provincial implementation.

**Conclusion:**

This paper has suggested several ways in which existing reforms may be leveraged to incorporate the objectives of the MHPF and achieve better mental health outcomes for South Africans, revealing critical opportunities for mental health service scale-up to be embedded in South Africa’s future health delivery strategy. The realization of a conditional grant for mental health will require technical expertise to cost existing services towards the development of an investment case for mental health service scale-up nationally, projecting potential resource requirements and returns on investment of a strong service platform. In the longer-term, the NHI benefit package must be expanded to include comprehensive mental health services at all levels. Explicit results-based financing mechanisms within the NHI Fund must also be incorporated for mental health to incentivise quality of care. Private providers engaged by the NHI must commit to make use of evidence-based mental health interventions.

## Background

Since a landmark publication by Prince et al. [1], the notion of “no health without mental health” has stimulated policy-makers in all countries to consider mental health and the treatment of mental disorders as a key priority in the pursuit of equity in health and health service access. This has become particularly pertinent in recent years with the emergence of universal health coverage goals and the need to provide broad-based, context- specific primary health care (PHC) [1, 2]. In 2018, the Lancet Commission on Global Mental Health and Sustainable Development reaffirmed and expanded these sentiments, emphasizing that a global response to mental health necessitates “promoting mental health, preventing mental disorders, and including mental health care in universal coverage…agenda[s]” as a humanitarian and development priority, providing evidence that mental health is indeed at the centre of sustainable development [3]. These goals have been embraced by the South African government through the adoption of the National Health Insurance Policy (2017) and the South African *National Mental Health Policy Framework and Strategic Plan* 2013–2020 (MHPF) [4, 5]. Despite compelling evidence supporting the case for investment in mental health systems and strong national policy commitments [6, 7], it is essential that the plans and policies developed to address the mental health burden in South Africa reflect an increased recognition that financing is a critical factor, not only in the realization of a viable mental health system but also for the long-term development prospects of the country.

Estimates from the Global Burden of Disease study (2016) have indicated that mental and substance-use disorders are the leading cause of Years Lost due to Disability (YLD) in South Africa. Estimates of Disability Adjusted Life Years (DALYs) and YLD attributable to Mental, Neurological and Substance Use (MNS) disorders represent 15.6% of all DALYs and 35% of YLDs due to Non-communicable diseases (NCDs) [8]. South Africa’s health system comprises a large public sector that serves about 80–85% of the population and a smaller private sector which is expanding rapidly. Considering that only 48% of the total health expenditure in South Africa is funded by the state, with the remainder being funded by the private sector, with 80–85% of the population relying on the state—the public health system is under extreme pressure to more effectively manage chronic, long-term care, while maintaining and improving the capacity of acute care services and addressing the challenges emanating from erratic medicines supply and sufficient health workforce [9]. Inequities in access to mental health care endure as a growing concern between Provinces, districts and among local communities. The limited resources that exist are inefficiently concentrated in large psychiatric hospitals, specializing in the treatment of severe mental disorder, with a predominantly vertical (disease focused, as opposed to integrated) model of care [10].

In 2011, the National Department of Health (NDOH) initiated a process of establishing a National Health Insurance (NHI) scheme to promote equity in health service delivery towards universal coverage [5, 11–14]. By design, the National Health Insurance model seeks to provide health care for all, irrespective of affordability and income band, and will be mandatory for all South Africans. Complete implementation of the NHI is set for 2025, and is set to be funded through payroll taxes, surcharges on taxable income and possible increased VAT revenues [5, 11, 15].

While the NHI efforts are ongoing, the NDOH has also made an explicit pledge to transform mental health services and ensure that “quality mental health services are accessible, equitable, comprehensive and are integrated at all levels of the health system” [4]; this commitment is reflected in the South African *National Mental Health Policy Framework and Strategic Plan* (MHPF), adopted in July 2013 [4]. The policy was intended to be fully realized by 2020 and envisages the complete integration of mental health care into general health services. As 2020 approaches, it has become apparent that there have been critical challenges in the implementation of the Plan, with no budgets dedicated to support its’ implementation. There is concern that if the South African mental health priorities are not explicitly addressed and reflected in the policies and activities supporting the overall implementation of the NHI, mental health is likely to continue to be relegated to the ‘backburner’, making the MHPF difficult to implement and the future prospects for the South African mental health system very uncertain [10].

With the implicit neglect for the integration of mental health services into general health service development in South Africa, there is an urgent need for an understanding of the ways in which existing reforms may be leveraged to incorporate the objectives of the MHPF and achieve better mental health outcomes for South Africans and more specifically, the mechanisms by which these reforms can be structured and financed in the context of fiscal constraint. This paper seeks to present the results of a situational analysis of the policy context, strategic needs, barriers and opportunities for sustainable financing for mental health in South Africa that is complimented with a synthesis of key stakeholder consultations. The findings seek to provide recommendations for how scaled-up mental health services can best be paid for in a way that is feasible, fair and appropriate within the fiscal constraints and structures of the country.

## Methods

### Study design

This study forms part of the Emerald (Emerging mental health systems in low- and middle-income countries) project [16], which was conducted across six low- and middle-income countries (Ethiopia, India, Nepal, Nigeria, South Africa and Uganda) and pursued a range of investigations into a number of mental health system strengthening components. Informed by similar frameworks developed for other disease priorities in the health sector, the Emerald project developed a new, stepped analytical framework for sustainable mental health financing [17]. The framework is structured around six domains: (1) assessment of the public health consequences of mental disorders; (2) assessment of the private and public economic consequences of mental disorders; (3) assessment of projected resource needs for scaling-up mental health services; (4) assessment of the mental health and general health system; (5) assessment of the current and projected macro-fiscal situation, and; (6) assessment and selection of appropriate financing mechanisms [17].

The results of the first three domains of this framework have been reported elsewhere [17, 18]. This analysis therefore seeks to report on the three remaining domains and was conducted in two parts. We first completed a situational analysis followed by a qualitative study involving in-depth, semi-structured interviews. The situational analysis served to address domain 4 and 5, specifically, whilst the in-depth interviews sought to compliment and validate the results of the document review and elicit responses linked to the all three domains (4, 5 and 6). These inputs were synthesized and fed into the development of financing recommendations for mental health service provision in South Africa in line with the health financing structure of the country and the policy directives of the NHI.

### Data collection

#### Situational analysis

The situational analysis was conducted in 2017 and updated in 2018. Online and printed data, grey literature, and government documents and policies were searched and reviewed to understand disease burden, health policies and plans, macro-fiscal and political context, as well as health-system governance and management of mental health care services in South Africa. In order to complete the situational analysis, documents relevant to general health services delivery such as policy and strategic framework reports, peer-reviewed articles, and other grey literature were obtained from the Department of Health (DOH) websites, World Bank Database, and reports and articles using key term searches. Further documents were obtained and reviewed upon recommendations provided during stakeholder interviews.

#### Qualitative in-depth interviews

Semi-structured Mental Health Financing Diagnostic interview guides were developed for each category of respondent to compliment the document review and elicit responses linked to the final three domains [19]. The interview guides covered a range of topics that explored the current conditions of the health and public sector; priority given to mental health (domain 4); ongoing health financing efforts and future plans as well as the budgetary and efficiency implications for mental health service development (domain 5); the main perceived challenges to increased public health financing and options for change required for sustained resources for a scaled up mental health service in South Africa (domain 6).

The sampling of respondents for the qualitative interviews was purposive, with a view to ensuring that the perspectives of health, policy and financing experts were obtained and to facilitate a participatory, consensus-building approach towards the development of recommendations [17]. Participants from a number of key sectors, including the NDOH and National Treasury (NT) were sampled, in addition to NGO respondents from the South African Depression and Anxiety Group (SADAG) and the South African Federation for Mental Health as well as a senior public sector researcher specializing in health financing at the University of Cape Town. The interviews were conducted in-person or telephonically and lasted an average of 1 h. Interviews were audio-recorded with informed consent from the respondent.

### Data analysis

The audio recorded interviews were transcribed verbatim and a framework analysis approach was used to analyse the qualitative data using NVivo 11 [20]. An a priori coding framework linked to the last three domains was developed to structure and summarize the responses.

## Results

Table 1 (below) summarizes the number of stakeholder interviews that took place and the organizational affiliations of each respondent. Two interviewees were affiliated to the NGO sector, one was affiliated to the National Department(s) of Health, one from the National Treasury, with the last stakeholder affiliated to an academic institution with public health financing expertise.

**Table 1:** Stakeholder interviewee descriptions

Total stakeholders interviewed	Organization affiliations
1	National Treasury
1	National Department of Health: Non-communicable Diseases
1	NGO Sector: South African Depression and Anxiety Group
1	NGO Sector: South African Federation for Mental Health
1	Academic Research Institution (Public Health Financing specialist)

### Macro fiscal and Health-system context

Since 2012 public-health expenditure has only increased by an estimated 1.8% per year [11, 21], with expenditure on health currently at 13.5% of total government expenditure and unlikely to reach the Abuja target of 15% [11, 21] This is coupled with marginal economic growth of 1.3% in 2017 [22] and low growth projections forecasted until at least 2020. These figures are concerning in the face of a growing population of uninsured South Africans who rely on the public health system, rising by an estimated 1.52% per year [11]. According to Statistics South Africa (STATS SA), 86% of the total spend on healthcare is spent by provincial governments, tasked to manage the nation’s public healthcare system, comprising of 422 hospitals and 3841 clinics and health centres [23]. The main expenditure items were hospital services (62%), public health family planning and disease detection (33%), and ambulance services (4%) [23]. The in-depth interviews raised a number of concerns with respect to how the government has contained costs in the health sector following the economic recession, using strategies such as: limiting personnel numbers, centralised tendering for medicines and delays in major capital projects.

Further adding to this pressure is the implementation of *National Health Insurance for South Africa (*NHI) [5, 24] which represents an upward trajectory for health expenditure in the face of fiscal constraint [11]. There is concern over the way in which the NHI pilots have been run and many stakeholders believe that it has been a wasted opportunity. Of note is the exclusion of key constituencies participating in NHI implementation including community health worker and nurses at the expense of powerful private sector bodies leading the process. As explained by a public financing expert:

*“I’m very disappointed at how the NHI pilots have been run… they’ve been focusing on interventions related to maternal and child health. Why is there not a psychiatrist in the clinical specialist teams …because [mental health] doesn’t have as direct an impact on mortality and I think it’s been an absolutely wasted opportunity….to deliver comprehensive services that actually address the whole range of issues”.*

Despite the adoption of the South African MHPF (2013), health budgets and broader health sector transformations have not followed to actualize the contents of the policy [4, 25]. Most critically, there remains a lack of consistency between the content of, and priorities outlined in the MHPF and those expressed in the NHI Policy [13, 26, 27]. The Guidelines have included mental health in the comprehensive package of services being re-engineered in the primary health system, and have specified the work of the Community Health Workers (CHWs) to include psychosocial support, adherence support for chronic conditions and referral support to social and health services. However, the training programs and manuals that have been developed and rolled-out for both for the CHWs and their team leaders have not included training on mental health [27–29] and up until 2015, households profiled by the outreach teams reported no assessments or referrals for mental health. [28].

Similarly, the implementation of the Integrated School Health Policy (ISHP) introduced in 2012 has neglected mental health service provision [30]. Notably, across all of the ten pilot sites, the ISHP did not identify a single learner with mental illness or substance use disorder, despite the inclusion of the identification of cognitive and related developmental impairment in the range of services provided by the ISHP, among numerous other mental health services [28, 30].

There are a multitude of factors that have weakened the provision of mental health services in South Africa, most critically the lack of human and financial resources to address treatment gaps [10, 31, 32], limited routine information systems to understand the true burden of mental disorders and utilisation patterns and high levels of stigma [33] As a result of poor access to good quality primary mental health care, the entry level for accessing mental health services at present is mostly at an inappropriate level of care (tertiary and specialist psychiatric services) [10, 27]. This has significantly contributed to the high costs of health care and the inefficiency of the health system [27]. This has also meant that care-seeking typically occurs when patients experience very severe symptoms, largely as a result of untreated mental illness, and often require long-term institutionalized care. Approximately two thirds of discharged patients (from psychiatric facilities) are readmitted, and largely remain institutionalized without much potential of returning to their communities [34, 35]. This is largely due to limited availability of well-resourced community-based residential and day care service to manage mental health care users after discharge, coupled with the impact of poverty on households, with many families unable or unwilling to care for family members after discharge [36].

While in-depth interviews acknowledged the significance of the policy effort, the key blockage has been a lack of budget allocations at the provincial level to allow for the implementation of the MHPF. As one interviewee explained:

*“…without explicit earmarking of funds there is no way of guaranteeing the actualization of the MHPF, particularly when considering South Africa’s decentralized fiscal system and the current environment of strained fiscal capacity”.*

Most stakeholders also acknowledged the biggest hurdle has been at provincial implementation, and the very institutionalized model of mental health care continues to persist with services and resources concentrated in hospitals. As clarified by one respondent from the NGO sector:

*“…we find that…implementation in terms of the provinces…we can’t get any go*-*ahead, even if there’s policies at National or buy*-*in from National…provincial implementation is where the blockages are…so it doesn’t really help to have a policy”.*

Similarly, a public financing expert reaffirmed this view clarifying that:

*“…the vast majority of money for health services comes through provinces. And your battleground is every single province… who are currently struggling to actively fund existing services”.*

Both the MHPF and the Mental Health Care Act (MHCA) (2002) explicitly mandate the role of the district hospital as the first point of contact for mental health care users (MHCUs), and assigns the responsibility of ensuring that MHCUs are assessed and provided with ongoing referrals to more specialist treatment within a 72-h period [4, 37, 38]. Presently however, the majority of district hospitals in the country are not equipped with the infrastructure required to safely admit MHCUs for a 72-h observation, nor are they equipped with adequate room space for group therapy and self-help groups or workshop space for occupational therapy, as mandated by the MHCA [4, 39]. Further, the White Paper on the NHI (2015), has specifically excluded Psychiatry and/or Mental Health services from the four cited disciplines on work to be provided at the District hospital [27, 39]. This is in contradiction to efforts to integrate mental health services at lower level services to ensure wider access to care with district hospitals meant to serve as the first point of care for mental health care users.

Stakeholders mentioned that there has been little capacity for District Management structures to engage with mental health issues. The current tiered structure of the healthcare system, and the commitment to greater autonomy at the district level, in the context of ambitious reforms such as the NHI—“has created a situation wherein the success or failure of healthcare reforms will largely revolve around the strengths and weaknesses of district management” [40]. As one stakeholder noted:

“…[they] don’t have the knowledge of what they are required to deliver…there are no measurable deliverables” associated with the MHPF for the district level, making it a very low priority for overburdened district health management teams”

### Challenges to increased public and mental health financing

There is a lack of data available for mental health financing in South Africa, with Government being the main source of funding through tax-based health budgets [41]. Provincial and national budgets for mental health services are not reported or routinely available. Based on modelled estimates (for expenditure on psychiatric hospital level services only), South Africa spent an estimated $59 million (US$ 5.94 per capita [41]) on mental health services in 2005 [42]. The National Government of South Africa uses two types of transfers, conditional grants and unconditional provincial equitable share funds, to send money to provinces in South Africa. Presently, South Africa does not have a ring-fenced budget for mental health and funding falls under general health allocations of the equitable share. This means that provinces receive a set amount of funds from the national revenue, based on a provincial equitable share formula, and resource allocations to health and to specific health programmes are therefore determined by the Province’s own priorities.

While this approach to financing is consistent with global trends of decentralizing expenditure responsibilities, stakeholders felt that it has contributed to a situation in which increases in resourcing to Provinces do not guarantee use of these resources for their intended purpose, and these provincial decisions often redirect additional resources for health to other needs. Provinces are not required to report on expenditure for specific health programmes paid for through the equitable share transfer, making it difficult to assess whether Provincial budget priorities are aligned to National priorities for health. Stakeholders from all sectors believed that motivating for mental health to be included in provincial equitable share is therefore unlikely to yield any measurable increases in revenues for mental health, or any measurable improvements in the mental health system.

Further, the absence of ring-fenced allocations for the development and maintenance of the specialized psychiatric service at the tertiary level has created a number of substantial challenges: the psychiatric hospitals are outdated and in disrepair; there is an acute shortage of mental health professionals available to deliver this service; these facilities are unable to invest in the advancement of their scope of service (for example, child and adolescent psychiatry, neuropsychiatry and old age psychiatry) [34].

The NGO sector also reported severe challenges with respect to financing and it was estimated that 50% of the mental health NGOs in South Africa are struggling with sustainability at present. To secure funding from the Department of Social Development and/or Health, most mental health NGOs in South Africa have needed to commit to the delivery of statuary interventions, not mental health services. The NGO stakeholders believe that due to these models of funding, their mental health services become diluted and unspecialized—focusing on family planning, or foster care, or services to the aged.

### Options for change for a scaled up mental health service in South Africa

#### Budget planning and allocations for mental health

The process of health budgeting has changed in the past 10 years, motivated by increased pressure after the 2008 recession and increased complaints around fiscal federalism and the lack of control over the use of funds by the Minister of Health. The respondent from the NT explained:

“…increasingly, rather than just giving an unlinked equitable share increase and allowing provinces to decide where to allocate these increases, the role of NT and the National Process has become more prominent…increases in budgets for major changes are hinged on the capacity and technical expertise that program managers possess in order to put together an effective budget bid…one shouldn’t be too pessimistic about funding possibilities….we have funded many things…[but] we’ve had very few mental health budget bids…a lot of programs don’t have economic capacity… they know what they want to do, but they don’t quite know how to convert it into a plan and cost it…”.

Recently, the NT funded an HIV and TB investment case, representing the first time any HIV and TB investment case was funded. A finance-level state stakeholder suggested that there is reason to be optimistic and that despite prevailing opinions being that there is simply not enough money, there are funds that could be made available if mental health tabled a series of big budget bids as was done for TB and HIV. Should a budget bid for mental health be successful, it would ensure an escalating resource envelope for mental health. As one respondent explained:

“…we’d have to tell provinces, “this money is for doing the following” …and the more specific…detailed and measurable it can be…in terms of the way you’ve costed it, the easier it is for us to know whether the provinces are using the money for that.”

The likelihood of seeing a successful budget bid for mental health was challenged somewhat by the health-sector stakeholder, who believed that demonstrating cost-saving and returns on investment for mental health does not necessarily guarantee it will be funded:

“…even if we can demonstrate that [investing in] mental health makes sense…in this climate …unless something very dramatic happens economically in our country, and that doesn’t look very likely at this point in time…there isn’t going to be a lot more resources to give around…what one really needs to be looking at more is “how does one make better use of resources” rather than “how do we get more resources”.

Nonetheless, the respondent from the NT did also mention that a key criterion for evaluating these bids is the ability to demonstrate efficiency gains and value for money, meaning that efficiency is a priority for both health and the finance-sectors. Some opportunities for improving efficiency that emerged from the interviews included: improved matching of human resource posts and budget with workload; a review of hospital platforms with activities that support shorter length of stays and greater outpatient care, and; reducing budgets for new facilities, with a focus on dedicating budgets to ensure existing facilities that are only partially operationalized become fully operational.

A further difficulty to the budget planning process was identified as the very medical model used by the DOH, and the difficulty in conceptualizing developmental models—which leads to very little resourcing for psychosocial rehabilitation, outside of the licensing of these facilities with most resources going directly into hospitals. Yet this service is a critical component of treatment for the service users. Respondents noted the need for increased advocacy among policy makers to ensure that both developmental and medical models of mental health care are recognised, with adequate recognition and resources dedicated to community psychosocial support.

At present, stakeholders reported that there is no way of monitoring mental health financing in the public sector outside of specialized hospital care for mental health. The NHI model is intended to include improved expenditure tracking and mechanisms by which the NHI Fund will be able to associate services at all levels of the health care system with the resources made available, however these mechanisms are still not fully developed and little information has been provided to these stakeholders regarding how and when this improved monitoring system will be implemented. It is hoped that this will ensure the health system can be more responsive and accountable.

#### Strengthening mental health systems

Stakeholders who were interviewed highlighted a series of mental health system reforms that should be prioritized. These included: the explicit inclusion of mental health services in NHI implementation efforts through the expansion of the mandate of District (first referral) hospitals to include mental health services in their priority services; secondly, investing in infrastructure upgrades required to safely treat patients who are admitted for 72-h observation, as per the MHCA (2002); ensuring the availability of mental health specialist staff in District and community health services whilst allowing for psychiatrist input into district management teams and ensuring that clinical specialist teams include at least one psychiatrist; and investing in targeted mental health training for all generalist staff particularly in mental health screening and diagnosis including anti-stigma training. Due to the critical shortage of psychiatrists working in the public sector, this strategy would necessitate contracting of private providers and the provision of an explicit reorientation program to ensure they commit to the delivery of therapies based on a public health approach, and; acknowledgment that particularly in rural settings, a Medical Officer with a Diploma or an interest in psychiatry may be the only available option in the short-term.

The potential for the private sector to address the chronic human resource shortage for mental health services in the public sector was also emphasized by a number of stakeholders. Considering the discussions around the NHI, accessing private sector resources, including human resources, would be facilitated through contracting the private providers through the NHI central fund.

“…when you look at how much is being spent in the private sector and how many people are in the private sector dealing with mental health, it’s pretty substantial… if we could unlock all of those people… …if they moved away from, sort of doing long-term therapies to doing more community-oriented work and to looking more at preventive interventions and so on, there’s huge resource”.

There was consensus that there is interest from private sector psychiatrists and psychologists to contribute their services and time to the public sector, however challenges have already emerged with respect to the provider payment contracting through the NHI implementation efforts, with provinces not having the resources to pay the private providers for their time. Details of the strategic purchasing arrangements through the NHI Fund are still unclear, however would impact on the success of drawing on the private sector. As one respondent explained:

“…[only] on their terms…where and how their services are provided and also at what price…and that’s not affordable”.

The interviews also emphasized the need for an integrated multi-sectoral response to community-based service delivery for mental health:

“…we have education, we have health, we have social development, we have public works, we have transport and all these must come into one package for mental health services, because only then we have adequate resources”.

According to the respondents, strengthening community-based service delivery must to be complimented with an empowerment of mental health care users through education, particularly for those with intellectual disabilities and severe mental health problems. In addition, all stakeholders believed there is a need to engage with communities to establish their own needs, and to understand the range of NGOs operating within their districts to ensure a more efficient and targeted model of service. There was a strong importance placed on capitalizing on the capacities of all stakeholders involved in mental health service delivery into a unified, efficient service.

#### Financing mechanisms

Innovative financing mechanisms do not serve as a significant revenue generation source for the health sector, and although these mechanisms are exciting and innovative, their contributions are marginal in the broader sense, according to the respondent from the NT:

“…so called “innovative financing” is a bit fringe in a way…that sits on the margin… it doesn’t really matter whether funding is raised through VAT or personal income tax or company tax, or alcohol tax or tobacco tax … in general the source of revenue is not so relevant for the health sector…what’s important is that there is sufficient revenue for the service”.

On the other hand, while conditional grants from the NDOH make up only 20% of provincial health department budgets, they play a very important role in provincial health care delivery because are used by the National government to protect special health programmes or start up new programmes. This commitment over the next several years could ensure that Provincial departments submit detailed business plans for the allocation of funds to various mental health systems activities, aligned with performance targets detailed in the MHPF. The NDOH would then be responsible for approving these business plans and transferring funds to provincial or local departments for their implementation. This mechanism will require that Provinces and local governments report on their expenditure against specific mental health targets. In the short term, the priority areas for activities funded through a conditional grant should include the development of community based mental health services in South Africa.

In the long term, the transition to the NHI model was highlighted as the main mechanism to generate additional funding for health and could potentially play a key role in reversing the trend of low public health expenditure growth. NHI should increase public funding from around 4% of GDP to around 6% of GDP; the NHI is considered the best chance for increased funding for the health sector and further provides an opportunity for the provinces to purchase services from the private sector.

## Discussion

This paper set out to synthesize new evidence and perspectives relating to the current policy context, strategic needs, and opportunities for mental health resourcing in South Africa with a view to providing recommendations for how scaled-up mental health services can best be paid for in a way that is feasible, fair and appropriate within the fiscal constraints and structures of the country, and in line with the transformation of the health sector toward NHI. Despite the country’s comprehensive MHPF, progress in service delivery is challenged by a combination of weak health information systems to understand the true burden of disease, inequitable health service access due to the legacy of the apartheid system, ongoing inequities in economic and employment opportunities, macro-fiscal strain and multiple competing health priorities in an environment of reduced fiscal capacity and a growing population with chronic health needs. Early evidence from NHI pilot districts point to discordance with the MHPF and limited integration of mental health service provision in the country’s PHC strengthening plans, highlighting a significant missed opportunity for sustainable mental health services in the long term [43]. Plans around the NHI are intended to move South Africa closer to achieving UHC; this cannot be achieved in the absence of the explicit inclusion of integrated mental health service planning and sustainable resourcing. Mental health remains an integral component of health care, both in light of the significant and growing burden of MNS disorders and the high level of comorbidities with other major conditions, as well as its impact on overall population well-being. Improved and sustainable mental health financing to improve access to care for all citizens remains a fundamental human right and is aligned to the global Sustainable Development Goal of ensuring health and wellbeing for all; and aspiring to actualize a world, in which “physical, mental and social wellbeing are assured” [44].

Better managing the country’s existing health resources, advocating for the increased decentralization of health system resources to primary and community-level mental health care, in addition to intersectoral collaboration to address the upstream determinants of mental health conditions, ensuring earmarked funding for mental health in in the short-term and the explicit integration of mental health plans in the NHI efforts were recommended as efficient and sustainable approaches to scaling-up the South African mental health service and its’ financing. This is consistent with the recommendations of a joint initiative of the World Bank and World Health Organization (WHO) which emphasized the need for a multi-sectoral, global response to mental health as a humanitarian and development priority [45]. The event culminated with a global investment case for mental health, clearly outlining how equitable investments in primary and community-based mental health systems can lead to clear and definable health, economic and social benefits [45, 46].

The study revealed that improving the management of the country’s existing resources may entail: decentralising and deinstitutionalizing services once an effective community-based platform for mental health service delivery is established to reduce hospital length of stays and readmission rates by strengthening the transition between hospital care and the next source of care within the community [47]; task-shifting and task sharing approaches including nurse-initiated psychotropic medication [48] and the explicit integration of mental health care into chronic care services at all levels of the health system [49]. In addition, these efforts should be implemented in parallel with the implementation of more effective information systems with concrete Provincial deliverables in place to monitor implementation.

The creation of a mental health conditional grant in the short- to medium-term emerged as a critical recommendation for ensuring a stable funding source is in place to reverse historical trends of budgeting for mental health, and ensure parity in financing with other health priorities in the country. Key challenges to successful budget bids for mental health were identified as the lack of technical expertise to convert the activities outlined in the MHPF into measurable and specific plans that quantify the financial costs and the yields on the investment, and; the difficulty in quantifying the population level outcomes as a result of significant new investments in mental health care. Therefore, advocating for this conditional grant will require the NDOH to source technical expertise to systematically cost the existing mental health service; and use these cost estimates to develop an investment case for mental health service scale-up nationally, projecting the potential resource requirements and returns on investment of a strong mental health service platform, by province and across geographies. Following the Life Esidimeni tragedy in South Africa [50], there is a strong level of political will for mental health service strengthening at present, and thus a conditional grant for mental health in the short-medium term will capitalize on the political appetite for change, and lead to a sustained focus on mental health as the country engages in broader health sector strengthening efforts.

Once in place, the priority should be to invest in developmental models of care by strengthening the community based mental health service, which is largely non-existent and fragmented [48]. This must include infrastructural investments in home-based and community residential care facilities, while maintaining current financing of specialized psychiatric hospitals already included in the provincial equitable share; psychiatric hospitals being the only level of mental health care for with a dedicated line item budget is specified within provincial budgets, further reinforcing a hospi-centric model of care. This will also include financing the provision of training for CHWs, PHC nurses and generalists working in the district health sector and ensuring that funds are dedicated to obtaining population-based estimates of the prevalence of mental health disorders.

In the longer-term, study findings support the recommendation that mental health is included in general health resource development focussed on raising public funds through the implementation of a NHI system, by expanding the NHI benefit package to explicitly include comprehensive mental health services at all levels of the health system, as outlined in the MHPF with priority given to community-based mental health services. As the South African government moves toward developing the exact mechanisms by which the NHI Fund will operate and pay providers, we recommend that the government includes results-based financing as a key feature of the NHI provider payment mechanism and ensures that performance targets for mental health specifically are included and therefore incentivised. Results-based financing can improve efficiency by offering high remuneration for services performed at PHC centres, particularly for early and continued community support and referrals for severe mental disorders [51], which will reduce the burden on specialized hospital based services. Results based financing also has the capacity to improve the quality of care [52], and may catalyse a reduction in the stigma that mental health care users face in accessing care, particularly at lower levels of the health care system.

Finally, we recommend that the well-developed private mental health sector in South Africa is leveraged through contracts with private providers. This may be a means of improving coverage of mental health services in hard to reach and/or underserved areas of the country and ensure that quality clauses for mental health services are explicitly outlined in these contracts [53]. Funds to facilitate contracting with private providers should initially be covered by the conditional grant for mental health (to bypass current experiences where Provinces are not able to pay these providers), and once mechanisms are established for contracting with private providers through the NHI Fund (by 2025), these costs can be transferred to the NHI fund, in line with the long-term recommendation of including mental health in general resource development. Private providers should also be contracted to provide specialized psychiatric input to support district health management teams, and clinical support to primary care providers and generalists. This recommendation is not without some risks and is contingent upon ensuring that private providers who are engaged by the NHI make use of evidence-based mental health interventions that make optimal use of scarce public resources. Particularly in the field of psychological therapies, priority should be given to practices that are based on proven evidence of clinical benefit, and that adopt a public health approach.

Limitations of this study include the availability of current statistics and information pertaining to mental health service coverage and burden of disease. While the situational analysis did attempt to gather information from a large selection of both peer-reviewed and grey literature in addition to policy and strategic documents to provide a comprehensive picture of the political and economic backdrop of mental health service delivery; the small number of purposefully selected interviewees may limit the breadth of perspectives available to inform the synthesis of this paper.

This study builds on others reporting on mental health services in South Africa by offering explicit financing perspectives and recommendations for mental health, contextualized to South Africa’s ongoing NHI plans. Future areas of study that incorporate a wider stakeholder perspective, particularly the NGO sectors and increased cross-sectoral input from the Departments of Health and Education, may help to identify other opportunities for shared actions for improved efficiency in mental health service delivery. Furthermore, district level inputs to help further identify ground level implementation challenges around the NHI and the incorporation of mental health services into PHC strengthening efforts would support the re-orientation of strategies as the NHI enters its second stage of implementation.

## Conclusion

In a context of weak integration of mental health services into general health services in South Africa, this paper has suggested several ways in which existing reforms may be leveraged to incorporate the objectives of the MHPF and achieve better mental health outcomes for South Africans. Better managing the country’s existing health resources and advocating for the increased decentralization of resources to primary and community level mental health services have been outlined as strategies for more efficient financing mechanisms for an equitable system of mental health service delivery. Furthermore, active integration of mental health in the ongoing NHI implementation, the submission of costed budget bids for mental health, while ensuring that information systems are in place to monitor implementation provide a critical opportunity for mental health service scale-up to be embedded in South Africa’s future health delivery strategy.

## Abbreviations

CHW: community health workers
DALY: Disability Adjusted Life Years
DOH: Department of Health
EMERALD: emerging mental health systems in low- and middle-income countries
ISHP: Integrated School Health Policy
MHCA: Mental Health Care Act
MHCU: mental health care user
MHPF: National Mental Health Policy Framework and Strategic Plan
NCD: non-communicable disease
NDOH: National Department of Health
NGO: Non-Governmental Organization
NHI: National Health Insurance
NT: National Treasury
PHC: primary health care
SADAG: South African Depression and Anxiety Group
STATS-SA: Statistics South Africa
YLD: Years Lost due to Disability
